# Mindfulness-Based Cognitive Therapy for Anxiety, Depressive Symptoms and Psychological Distress in Women with Breast Cancer: A Systematic Review and Meta-Analysis of Randomised Controlled Trials

**DOI:** 10.3390/jpm16100515

**Published:** 2026-09-30

**Authors:** Joshuan J. Barboza, Alma D. Santiago-Mijangos, J. Smith Torres-Roman, Flor M. Luna-Victoria, Oriana Rivera-Lozada, Cesar Bonilla-Asalde, Analy Torres-Mejia

**Affiliations:** 1 Vicerrectorado de Investigación, Universidad Señor de Sipán, Chiclayo 14001, Peru; riveraoriana@uss.edu.pe (O.R.-L.); bonasal@gmail.com (C.B.-A.); 2 Facultad de Enfermería, Universidad Veracruzana, Veracruz 91000, Mexico; alsantiago@uv.mx; 3 Escuela de Posgrado, Universidad Continental, Lima 15046, Peru; jstorresroman@gmail.com; 4 Facultad de Enfermería, Universidad Nacional de Trujillo, Trujillo 13001, Peru; flunavictoria@unitru.edu.pe; 5 Facultad de Medicina, Universidad Nacional de San Antonio Abad del Cusco, Cusco 08000, Peru; 171143@unsaac.edu.pe

**Keywords:** breast cancer, mindfulness-based cognitive therapy, anxiety, depression, psychological distress, fear of cancer recurrence, psycho-oncology, personalised medicine, systematic review, meta-analysis

## Abstract

**Background/Objectives:** Psychological distress, anxiety, depressive symptoms and fear of cancer recurrence affect many women treated for breast cancer and often persist beyond active treatment. Mindfulness-based cognitive therapy (MBCT) is manualised and can be delivered in groups or over the internet, which makes it a candidate component of personalised supportive care, but its effect has not been quantified separately from that of other mindfulness programmes. We estimated the association between MBCT, including internet-delivered formats, and four psychological outcomes in adult women with breast cancer and rated the certainty of the evidence. **Methods:** This study represents a systematic review and meta-analysis of parallel-group randomised controlled trials, reported in accordance with the PRISMA 2020 statement. PubMed/MEDLINE, Scopus, Web of Science Core Collection and Embase were searched from inception without language restriction. The search was updated on 17 September 2026 in PubMed/MEDLINE, Scopus and Web of Science Core Collection; Embase, searched in the original search, could not be included in the update because its records could not be exported and institutional access subsequently ended; this is reported as a limitation. Trials were eligible when participants with breast cancer could be identified separately or, where they could not, when at least 90% of randomised participants were women with breast cancer, a threshold defined before data extraction. Two reviewers independently screened records, extracted post-intervention data directly from the published tables of each trial and appraised risk of bias with the Cochrane Risk of Bias 2 tool. We fitted random-effects models (restricted maximum likelihood); Hartung–Knapp intervals, fixed-effect estimates and a synthesis restricted to trials enrolling women with breast cancer only were sensitivity analyses defined before data extraction. Certainty was rated with GRADE. **Results:** Three trials (353 randomised participants, 340 of them women with breast cancer; one trial randomised 137 women with breast cancer and 13 men with prostate cancer and did not report results separately by tumour type) from Japan and Denmark were included; no outcome was informed by more than two trials. MBCT was associated with reductions in anxiety (Hedges’ g = −0.69, 95% CI −1.25 to −0.13; two trials, 182 participants) and in depressive symptoms (g = −0.90, 95% CI −1.46 to −0.35; two trials, 182 participants). Associations with total psychological distress (mean difference −5.09, 95% CI −10.20 to 0.02; two trials, 178 participants) and with fear of cancer recurrence, which rested on one trial, were imprecise and compatible with no benefit. Hartung–Knapp intervals included no effect for every outcome, and the anxiety and depressive-symptom analyses reduced to a single trial when the trial with a mixed cancer population was excluded, so neither apparent effect was robust to the method used to quantify uncertainty. Certainty was very low for all four outcomes. **Conclusions:** MBCT is associated with moderate-to-large short-term reductions in anxiety and depressive symptoms, but each estimate rests on two trials, is not robust to the inferential method used and is of very low certainty; three trials are too few to support firm recommendations, and the programme should be regarded as promising rather than established. Adequately powered trials with active comparators, common outcome measures and longer follow-up are needed. Routine checking for redundant publication is also required: one retrievable report, classified as a suspected redundant report on numerical comparison alone, presents data largely identical to those of an included trial.

## 1. Introduction

Breast cancer is the most frequently diagnosed cancer in women, with an estimated 2.3 million new cases and 666,000 deaths worldwide in 2022 [1]. Improvements in early detection and treatment have produced a large and growing population of women living after a breast cancer diagnosis, and with it a durable burden of psychological morbidity. Depressive symptoms and anxiety are more common in this group than in the general population and carry prognostic weight of their own: a synthesis of 282,203 patients linked both to higher recurrence and mortality [2]. Fear of cancer recurrence is the most frequently reported unmet need of cancer survivors, affecting roughly half of them at a clinically relevant level [3].

The clinical implication of that burden is not simply that psychological support should be offered, but that it should be targeted. Distress after breast cancer is heterogeneous in origin, trajectory and intensity, and the interventions available differ in cost, intensity and the skill mix they require. A personalised approach to supportive care therefore depends on knowing which structured psychological programme works, for which patient phenotype and in which delivery format, rather than on an average effect attributed to psychological care as a whole. Manualised group programmes are attractive within such a model precisely because they are transferable: they can be taught, supervised and audited, and they occupy a definable position between low-intensity psychoeducation and individual psychotherapy in a stepped and stratified care pathway. We note at the outset that an evidence base of the size available here can identify candidate patient and delivery characteristics but cannot establish which patient phenotype benefits most or which delivery format should be preferred; those questions are framed throughout this review as hypotheses for future trials rather than as conclusions.

Mindfulness-based cognitive therapy is one such programme. Developed to prevent depressive relapse [4], it combines mindfulness meditation with cognitive-behavioural elements and targets rumination, automatic negative thinking and experiential avoidance. Its eight-week structure has been adapted for oncology populations, and internet-delivered versions extend its reach to women who cannot attend group sessions because of distance, fatigue or the competing demands of treatment [5]. Recent trials of app-delivered mindfulness programmes show that this delivery route is feasible and can be cost-effective [6].

The evidence usually cited in support of mindfulness in breast cancer, however, does not speak to mindfulness-based cognitive therapy specifically. Existing syntheses pool it with mindfulness-based stress reduction and with a range of adapted programmes [7,8,9]. A recent stratified meta-analysis of 45 trials in adults with cancer makes the problem visible: the pooled effect for mindfulness-based interventions overall (g = −0.92 for depression) is substantially larger than the effect for mindfulness-based cognitive therapy considered on its own (g = −0.68), and modified programmes account for much of the difference [10]. Clinicians deciding whether to invest in an eight-week manualised programme need the estimate for that programme, not for the family it belongs to; this is the same stratification logic that governs treatment selection elsewhere in personalised medicine.

We therefore conducted a systematic review and meta-analysis restricted to randomised controlled trials of mindfulness-based cognitive therapy, including internet-delivered formats, in adult women with breast cancer, addressing four psychological outcomes defined before data extraction and grading the certainty of the resulting evidence.

## 2. Materials and Methods

### 2.1. Reporting and Registration

The review was conducted and reported in accordance with the PRISMA 2020 statement [11]; the completed checklist is provided as File S1. The review protocol was not prospectively registered in a public registry such as PROSPERO, OSF or INPLASY; the consequences of this are considered in Section 4.4.

### 2.2. Eligibility Criteria

Eligible studies were parallel-group randomised controlled trials enrolling adult women (≥18 years) with a confirmed diagnosis of breast cancer or a history of treatment for breast cancer. The intervention of interest was mindfulness-based cognitive therapy delivered in any format—group, individual, face-to-face or internet-delivered—compared with usual care, waiting-list control or no active intervention. Trials were required to report at least one of the four review outcomes as a continuous measure at a post-intervention assessment. Because trials in psycho-oncology are frequently conducted in mixed cancer populations, the population criterion was operationalised with an explicit threshold, defined before data extraction: a trial was eligible when breast cancer participants could be identified separately or, where they could not, when at least 90% of randomised participants were women with breast cancer. One included trial met the criterion by this second route, Nissen et al. [12], in which 137 of 150 randomised participants (91.3%) had been treated for breast cancer. The consequences of retaining a predominantly, rather than exclusively, breast cancer trial are addressed in a sensitivity analysis (Section 2.8), in the certainty assessment (Section 3.10) and in Section 4.4.

We excluded trials that combined mindfulness-based cognitive therapy with another active psychological intervention without an isolable arm; trials enrolling mixed cancer populations from which breast cancer participants could not be identified and in which fewer than 90% of randomised participants were women with breast cancer; single-arm, quasi-experimental and qualitative studies; conference abstracts; and trials in which pharmacological treatment of depression was the primary intervention. Where two or more reports described the same participants, only one was retained; the procedure used to identify such reports is described in Section 2.4.

### 2.3. Information Sources and Search Strategy

PubMed/MEDLINE, Scopus, Web of Science Core Collection and Embase were searched from inception, without language or date restriction; the dates of the original search and of the update are given below. The strategy combined controlled vocabulary and free-text terms for the population (breast cancer, breast neoplasms, survivors) with terms for the intervention (mindfulness-based cognitive therapy, MBCT, internet-based MBCT, iMBCT). The comparator was deliberately omitted to maximise sensitivity. Reference lists of included trials and of relevant systematic reviews were screened by hand. Full search strings for each database are given in File S2.

**Figure 1:**
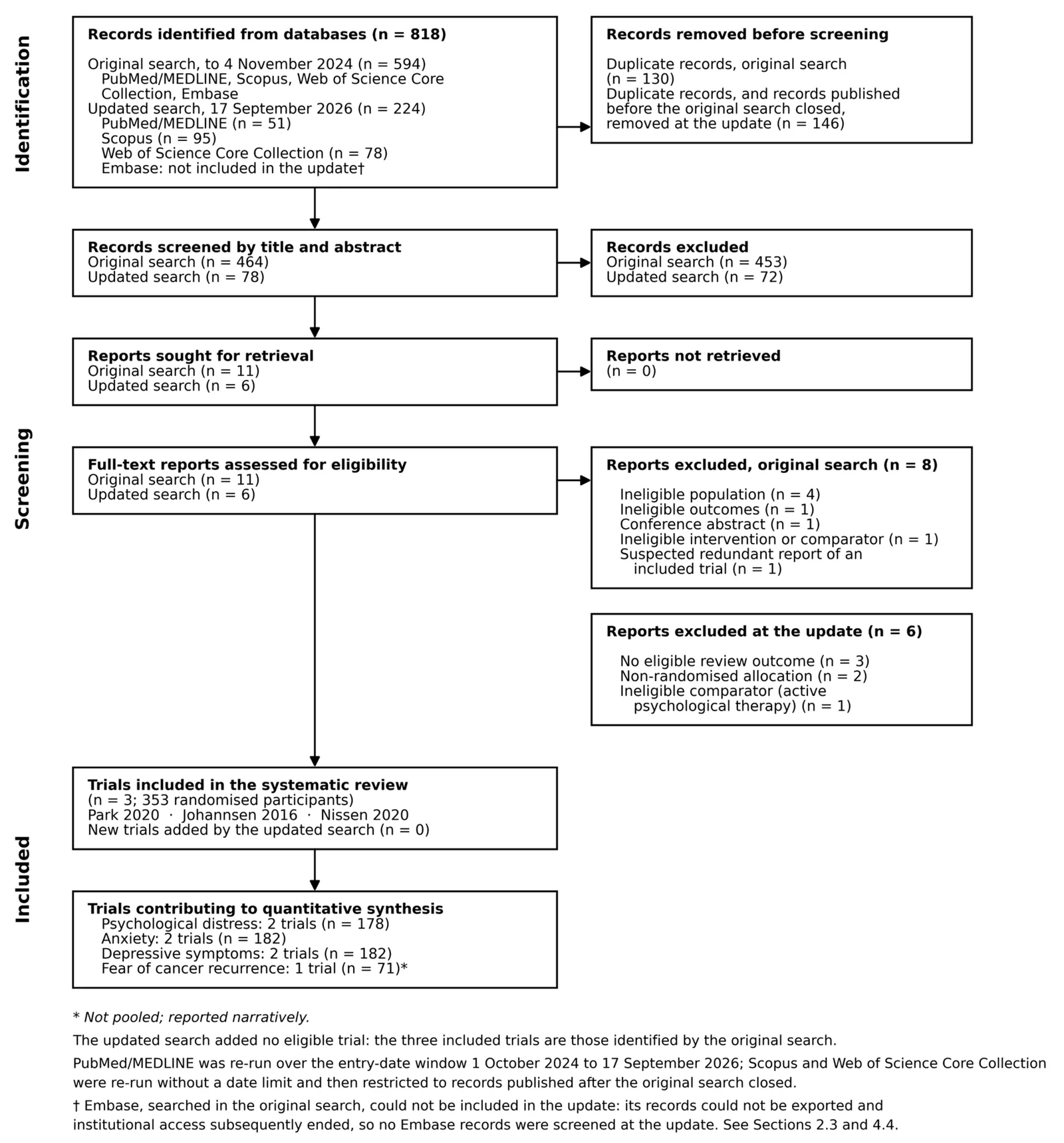
PRISMA 2020 flow diagram showing the flow of records through identification, screening and inclusion.

The original search was run to 4 November 2024. Because that date preceded submission by more than 18 months, and because an evidence base of three trials could be changed materially by a single additional trial, the search was updated rather than verified in a single database. On 17 September 2026, the strategy was re-run in PubMed/MEDLINE, over the entry date window from 1 October 2024 to 17 September 2026, and in Scopus and Web of Science Core Collection, the latter two being searched without a date limit so that the strategy could first be checked against the included trials and then restricted to records published after the original search closed. Embase, which had been searched in the original search, could not be included in the update. It was re-run on the same date and retrieved 76 records, but the platform would not export them, and institutional access to Embase ended before they could be retrieved and screened by any other route. The update therefore rests on PubMed/MEDLINE, Scopus and Web of Science Core Collection, and the unscreened Embase records are not counted in the flow of records in Figure 1. As a check on the sensitivity of the three databases used, the strategy retrieved all three included trials in both Scopus and Web of Science Core Collection. The consequences of the omission are considered in Section 4.4.

The three databases searched at the update yielded 224 records: 51 from PubMed/MEDLINE, 95 from Scopus and 78 from Web of Science Core Collection. Of the 47 Scopus and Web of Science records published in 2025 or 2026, ten were duplicated between the two databases, and ten of the remaining 37 were already among the PubMed/MEDLINE records, so the two citation databases added 27 records. After removal of duplicates and of records published before the original search closed, 78 records were screened by title and abstract, 72 were excluded, and six reports were assessed at full text. None met the eligibility criteria. Three of the six had been identified in PubMed/MEDLINE: a randomised trial of group mindfulness-based cognitive therapy against usual care in 45 women after mastectomy, which reported only sexual self-esteem and therefore contributed no review outcome; a secondary mediation and moderation analysis of a trial in 116 breast cancer survivors, in which the comparator was supportive-expressive sex education therapy, an active psychological intervention; and a study of 249 women with breast cancer that reported Hospital Anxiety and Depression Scale scores but allocated participants according to the treatment they chose rather than at random. Three were contributed by Scopus and Web of Science alone, all in journals not indexed in MEDLINE: a three-group study of mindfulness-based therapy that reported fear of cancer recurrence but described its design as quasi-experimental and reported no randomisation procedure; a study of fear of cancer progression and post-traumatic growth, which was also quasi-experimental and measured fear of progression rather than fear of recurrence; and a randomised trial of internet-delivered mindfulness-based cognitive therapy in 54 women that met every eligibility criterion except the outcome, reporting only pain intensity and quality of life. Records screened, full texts assessed and reasons for exclusion at the update are reported in File S2, and the search exports are archived with the analysis files.

Twenty of the 27 records added by Scopus and Web of Science were published in journals not indexed in MEDLINE. Those journals carry a substantial literature on mindfulness-based cognitive therapy in women with breast cancer, but, as the full-text assessments above illustrate, it is largely not randomised; the implication is taken up in Section 4.3.

### 2.4. Study Selection and Identification of Suspected Redundant Reports

Records were de-duplicated in EndNote 20 and screened in Rayyan. Two reviewers independently screened titles and abstracts and then full texts, with disagreements resolved by discussion and, where necessary, by a third reviewer.

Because psychological outcome data are frequently republished, each candidate report was additionally checked for redundancy against every other candidate. The check compared, cell by cell, the baseline and post-intervention means and standard deviations of all shared instruments, together with sample sizes, recruitment period and country. A report was classified as a suspected redundant report when a substantial proportion of cells were numerically identical to those of another report, on the reasoning that two independent trials are very unlikely to produce identical means and standard deviations to two decimal places across multiple instruments. The classification rests on numerical comparison alone: we did not obtain confirmation from the authors or the journals that the two reports describe the same participants or the same data set, and the term is used descriptively rather than as a finding of research misconduct. One report was excluded on this basis; the cell-by-cell comparison is reproduced in full in File S4.

### 2.5. Data Extraction

Two reviewers independently extracted data into a piloted form. Extracted variables covered study identification, country, design, sample size, participant characteristics, intervention delivery, comparator, follow-up, outcome instruments, analytical approach, adherence and attrition. Intervention characteristics were extracted against the TIDieR checklist [13] and are reported in Section 3.2.

For each outcome, we extracted the post-intervention mean, standard deviation and number of participants contributing to that mean, taken directly from the published tables of the source article. The exact table and page from which every value was taken are recorded in File S3, so that any reader can retrace each cell to its source. Where an instrument was reported only graphically, the trial was not entered into the corresponding analysis rather than having values estimated from a figure.

### 2.6. Outcomes

Four outcomes were defined before data extraction: total psychological distress, anxiety, depressive symptoms and fear of cancer recurrence. Because the included trials used different instruments, outcomes were defined by construct rather than by scale. Total psychological distress was measured by the Hospital Anxiety and Depression Scale total score [14]; anxiety by the anxiety subscale of that scale or by the state subscale of the State–Trait Anxiety Inventory; depressive symptoms by the depression subscale or by the Beck Depression Inventory-II; and fear of cancer recurrence by the Concerns About Recurrence Scale [15,16]. On all instruments, higher scores indicate greater symptom severity. Using the total score of the Hospital Anxiety and Depression Scale as a measure of general distress, rather than as the sum of two separable constructs, follows the empirical structure of that scale: a meta-confirmatory factor analysis of 28 samples found that a bifactor model in which all items load on a general distress factor fitted best, that the general factor accounted for 73% of the covariance between items, and that the scale is therefore best used as a measure of general distress rather than to separate anxiety from depression [17]. The same finding qualifies our use of the anxiety and depression subscales of that scale for the anxiety and depressive-symptom outcomes and is taken up in Section 4.4. For the one outcome expressed on a single instrument, the minimal important difference of the scale total score was taken as approximately 1.7 points, the value obtained in anchor-based analyses in patients with chronic obstructive pulmonary disease [18]. Because no minimal important difference for the scale total score has been established in women with breast cancer, this value is not specific to the population of this review; it is used only as a cautious contextual reference for the magnitude of the pooled mean difference and not as a threshold of clinical importance. No corresponding anchor was applied to the standardised outcomes, because a standardised mean difference pooled across different instruments cannot be referred to the minimal important difference of any one of them; magnitudes for those outcomes are therefore described in statistical terms only, and no claim of clinical importance is made for them.

### 2.7. Risk of Bias

Two reviewers independently assessed each trial with the Cochrane Risk of Bias 2 tool [19] across the five domains, at the level of the outcomes contributing to this review. Domain judgements were combined into an overall judgement of low risk, some concerns or high risk. Disagreements were resolved by consensus with a third reviewer. Domain-level judgements are reported in Section 3.4 and the supporting justifications are given in File S5.

### 2.8. Data Synthesis

Analyses were performed in R version 4.3.3 with the metafor package [20]. Effects were pooled with random-effects models fitted by restricted maximum likelihood. When trials contributing to an outcome used the same instrument, results are expressed as mean differences on that instrument, which preserves clinical interpretability; when they used different instruments, results are expressed as standardised mean differences with Hedges’ correction for small-sample bias. Heterogeneity was quantified with τ^2^, Cochran’s Q and I^2^.

Every outcome in this review was informed by at most two trials, which has three consequences that we handled explicitly rather than by default. First, τ^2^ is estimated with very little information, so I^2^ should be read as descriptive rather than as a stable estimate of inconsistency. Second, prediction intervals are not interpretable with two studies and are therefore not reported. Third, the Hartung–Knapp adjustment, which is generally preferable to the standard Wald interval when studies are few [21,22], rests on a t distribution with one degree of freedom in this setting and yields intervals so wide as to be uninformative. We therefore present the conventional random-effects Wald interval as the reference result, because it is the estimator used in the syntheses with which ours must be compared, and report alongside it both the Hartung–Knapp interval and a fixed-effect inverse-variance estimate (Section 3.9), so that readers can see the full range of defensible estimates rather than a single reassuring one. The wide Hartung–Knapp intervals are not treated here as an artefact to be set aside: they express the uncertainty that genuinely follows from estimating a between-study variance from two studies. No conclusion in this review is therefore drawn from statistical significance under the Wald model alone, and every pooled estimate whose Hartung–Knapp interval includes no effect is described as preliminary. With two trials per outcome, leave-one-out analysis reduces to the individual trial estimates, which are displayed in the forest plots. Publication bias was not assessed, since fewer than 10 trials contributed to any outcome. Outcomes informed by a single trial were not pooled and are reported narratively. A further sensitivity analysis, defined before data extraction, restricted the synthesis to trials that enrolled women with breast cancer exclusively, that is, excluding Nissen et al. [12]. Because that trial contributed to the anxiety and depressive-symptom outcomes, the restriction reduces both to the single estimate of Park et al. [23] and no pooling is possible; the restricted estimates are reported in Section 3.9 and their consequences for certainty in Section 3.10. The analysis script and the extracted data set are provided as File S6 and File S3, respectively.

### 2.9. Certainty of Evidence

Two reviewers rated the certainty of evidence for each outcome using GRADE [24], considering risk of bias, inconsistency, indirectness, imprecision and publication bias. Ratings and the reasons for each downgrade are presented in Section 3.10.

### 2.10. Use of Generative Artificial Intelligence

A generative artificial intelligence assistant was used to check numerical consistency between the manuscript, the analysis output and the source articles, to verify bibliographic records against PubMed and to improve the clarity and readability of the English language. No text, data, analysis or interpretation was generated autonomously and accepted without author verification. The search strategy, study selection, data extraction, risk-of-bias assessment, statistical analysis and GRADE rating were performed by the authors without the use of generative artificial intelligence.

## 3. Results

### 3.1. Study Selection

The original search retrieved 594 records; 130 duplicates were removed and 464 records were screened by title and abstract, of which 453 were excluded. Eleven full-text reports were assessed for eligibility and eight were excluded: four enrolled ineligible populations, one reported no eligible outcome, one was a conference abstract, one evaluated an ineligible intervention or comparator, and one was a suspected redundant report of an included trial. The updated search (Section 2.3) retrieved 224 records across the three databases searched at the update, of which 78 were screened by title and abstract after de-duplication, 72 were excluded and six were assessed at full text; none met the eligibility criteria. Three randomised controlled trials were included (Figure 1).

### 3.2. Characteristics of the Included Trials

The three included trials randomised 353 participants in total, of whom 340 (96.3%) were women with breast cancer (Table 1). Park et al. [23] randomised 74 Japanese outpatients with stage 0–III breast cancer to an eight-week group programme or a waiting list. Johannsen et al. [25] randomised 129 Danish women with persistent post-treatment pain to an eight-week group programme adapted for chronic pain or a waiting list; psychological distress was a secondary outcome. Nissen et al. [12] randomised 150 Danish cancer survivors screened for anxiety and depressive symptoms during routine oncology follow-up to a ten-week internet-delivered programme with asynchronous therapist support or to care as usual; 137 of the 150 randomised participants (91.3%) had been treated for breast cancer and 13 for prostate cancer, and results are not reported separately by tumour type; the trial was retained under the 90% threshold defined before data extraction (Section 2.2), and the consequences are reported in Section 3.9, Section 3.10 and Section 4.4.

**Table 1:** Characteristics of the three included randomised controlled trials.

Trial (Country)	Design and Setting	Participants Randomised	Mean Age (Years)	Intervention and Comparator	Outcomes Used in This Review
Park et al. [23] (Japan)	Single-centre, two-arm parallel RCT; tertiary hospital outpatient clinic, Tokyo	74 women (MBCT 38, waiting list 36); stage 0–III breast cancer; HADS total ≥ 5 at entry	MBCT 53.2 (SD 8.4); control 54.2 (SD 9.3)	Eight-week group MBCT, weekly 2 h sessions, daily home practice 20–45 min, meditation-guide CD, groups of 4–9; waiting-list control asked to refrain from mindfulness practice	HADS total; HADS-anxiety; HADS-depression; Concerns About Recurrence Scale (overall subscale)
Johannsen et al. [25] (Denmark)	Single-centre, two-arm parallel RCT; oncology department, Aarhus	129 women treated for primary breast cancer with persistent post-treatment pain ≥ 3/10 (MBCT 67, waiting list 62)	56.8 (whole sample)	Eight-week group MBCT adapted for chronic pain, groups of 13–17, daily 45 min home practice, single instructor; waiting-list control	HADS total score (secondary outcome of the trial)
Nissen et al. [12] (Denmark)	Single-centre, two-arm parallel RCT with 7:3 allocation; systematic screening at routine oncology and urology follow-up, Aarhus	150 cancer survivors with screening-detected anxiety or depressive symptoms (iMBCT 104, care as usual 46); 137 breast cancer, 13 prostate cancer	Breast cancer subgroup 54.6	Ten-week internet-delivered MBCT: eight modules of text and audio, weekly written tasks, asynchronous therapist feedback from supervised trainees; care-as-usual waiting list	State–Trait Anxiety Inventory (state subscale); Beck Depression Inventory-II

HADS, Hospital Anxiety and Depression Scale; iMBCT, internet-delivered mindfulness-based cognitive therapy; MBCT, mindfulness-based cognitive therapy; RCT, randomised controlled trial; SD, standard deviation. Nissen et al. [12] enrolled a mixed cancer population; the results are not reported separately by tumour type, and the whole-trial estimate is used here.

All three programmes retained the eight-module structure of the original protocol, with weekly sessions of about two hours in the face-to-face trials and daily home practice of 20–45 min. Facilitators were psychologists, psychiatrists and nurses with five to seven years of mindfulness experience in Park et al. [23], a mindfulness instructor with nine years of experience in Johannsen et al. [25], and psychology students supervised by an experienced psychologist in Nissen et al. [12]. Adherence was moderate: mean attendance was 6.8 of 8 sessions in Park et al. [23] and 5 of 8 in Johannsen et al. [25], while 56% of those who started the internet programme completed all eight modules in Nissen et al. [12]. Intervention characteristics are set out against the TIDieR checklist in Table 2.

**Table 2:** Description of the intervention in each trial against the TIDieR checklist [13].

TIDieR Item	Park et al. [23]	Johannsen et al. [25]	Nissen et al. [12]
1. Brief name	Mindfulness-based cognitive therapy for breast cancer	Mindfulness-based cognitive therapy adapted for post-treatment pain	Internet-delivered mindfulness-based cognitive therapy (iMBCT)
2. Why (rationale)	Reduce psychological distress, fear of recurrence and fatigue through metacognitive awareness	Reduce late post-treatment pain by altering the relationship to pain experience and reducing catastrophising	Extend access to mindfulness training for survivors with limited access to face-to-face treatment
3. What (materials)	Original MBCT manual with minor adaptation; meditation-guide compact disc; homework records	Manualised MBCT with pain-focused cognitive exercises; weekly homework records	Eight online modules with written and audio content; smartphone-compliant website; weekly task sheets
4. What (procedures)	Psychoeducation, formal meditation, compassion exercises and group discussion	Formal mindfulness exercises, psychoeducation, cognitive exercises targeting pain catastrophising	Module work with written assignments returned to the therapist; asynchronous written feedback
5. Who provided	Clinical psychologists, psychiatrists and nurses with 5–7 years of mindfulness experience, trained at the Oxford Mindfulness Centre	One mindfulness instructor trained at Oxford with 9 years of MBCT experience, supervised during the trial	Eight Master’s-level psychology students trained in MBCT and one experienced psychologist; students supervised by two authors
6. How	Face-to-face group sessions	Face-to-face group sessions	Fully online, therapist-assisted, individual
7. Where	Hospital outpatient setting, Tokyo	Hospital outpatient setting, Aarhus	Home; secure encrypted platform provided by a national internet psychiatry unit
8. When and how much	8 weekly sessions of 2 h; daily home practice 20–45 min; day-long retreat omitted	8 weekly sessions; daily home practice 45 min encouraged	10 weeks; 8 modules; mean 39 h 15 min total platform time; mean therapist time 117.7 min per participant
9. Tailoring	Brief cancer-specific psychoeducation added in session 1	Meditation exercises shortened; gentle yoga added; content oriented to pain	Content co-developed with a group of cancer survivors
10. Modifications	Retreat session removed as too burdensome for Japanese patients with cancer	Adapted from the standard protocol for a chronic pain population	Delivery format changed from face-to-face to online
11. How well (planned)	Therapists followed the session schedule; a research assistant observed sessions and checked adherence	Adherence monitored with weekly homework records	Module completion defined by return of a completed weekly task sheet
12. How well (actual)	Mean 6.8 of 8 sessions attended; mean homework of 24.2 min/day; 3 of 38 discontinued (7.9%)	Mean 5 of 8 sessions attended; mean homework 24 min/day; 31.3% did not return post-intervention questionnaires	82 of 104 (79%) started; 46 of 82 (56%) completed all eight modules; 49% did not start or discontinued within four weeks

TIDieR, Template for Intervention Description and Replication. Items are reported as described in each source article; blank or inferred information is not imputed.

### 3.3. Identification of a Suspected Redundant Report

One report retrieved at full-text screening, published in 2020 in a pharmaceutical sciences journal and describing 84 Chinese women, was excluded as a suspected redundant report of Park et al. [23]. Across the instruments the two reports share, a large proportion of cells are numerically identical to two decimal places, including the entire control-arm series for the Hospital Anxiety and Depression Scale total score at baseline, week 8 and week 12 and every reported value for the Functional Assessment of Cancer Therapy–General and the Five Facet Mindfulness Questionnaire. The report also states in its Results text a between-group difference in total scale score that does not match its own table but does match the value reported by Park et al. [23]. The full cell-by-cell comparison is given in File S4.

We record this because the report is retrievable through several databases, is not indexed in PubMed, and has been treated as an independent trial in at least one previous synthesis. Reviews that include both reports pool the same participants twice, which inflates precision and can shift a pooled estimate materially. We describe the report as a suspected redundant report rather than as an established duplicate publication because we could not obtain direct confirmation that the two reports describe the same participants or the same data set. The inference rests on the numerical comparison reproduced in File S4 and on the implausibility of the agreement it shows; we have not approached the authors or the journals, and we make no claim about how the duplication arose.

### 3.4. Risk of Bias

Park et al. [23] was judged to raise some concerns overall: sequence generation and allocation were handled by an independent clinical research centre, but participants could not be blinded to a group programme, outcomes were self-reported, and no protocol registration was reported. Nissen et al. [12] was also judged to raise some concerns, driven by an unbalanced allocation ratio with the sequence generated by the primary investigator and by attrition of 26–31% in the intervention arm; the trial was prospectively registered and used intention-to-treat multilevel modelling with sensitivity analyses for missing data. Johannsen et al. [25] was judged to be at high risk of bias, principally because 31.3% of the intervention arm and 1.6% of the control arm failed to return post-intervention questionnaires, a differential that the authors themselves flag. Domain-level judgements are shown in Table 3.

### 3.5. Psychological Distress

**Figure 2:**
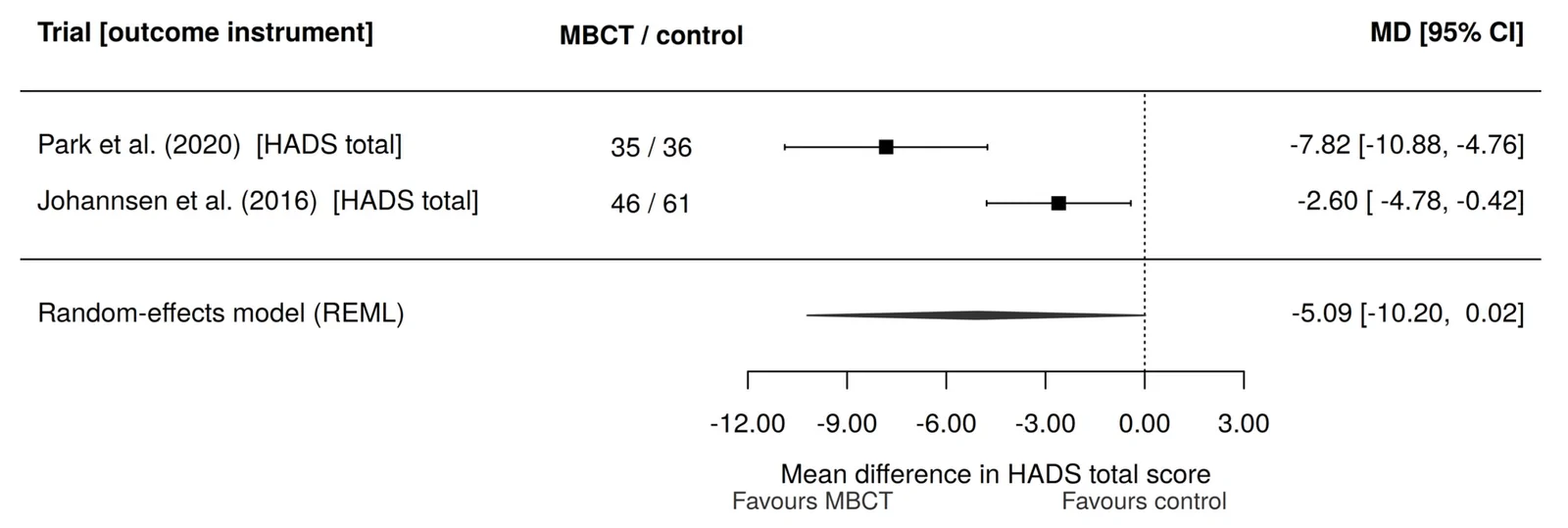
Forest plot of the association between mindfulness-based cognitive therapy and total psychological distress (Hospital Anxiety and Depression Scale total score). Random-effects model, restricted maximum likelihood. Squares are trial mean differences with 95% confidence intervals, sized by weight; the diamond is the pooled estimate [23,25].

Two trials measured total psychological distress on the Hospital Anxiety and Depression Scale (178 participants). The pooled mean difference favoured mindfulness-based cognitive therapy but was imprecise and compatible with no effect (−5.09 points, 95% CI −10.20 to 0.02; Figure 2). Heterogeneity was substantial (*I*^2^ = 87%, *τ*^2^ = 11.78, *Q* = 7.40, *p* = 0.007), and the two trial estimates do not overlap: Park et al. [23] reported a difference of −7.82 points (95% CI −10.88 to −4.76) and Johannsen et al. [25] a difference of −2.60 points (95% CI −4.78 to −0.42). The contrast is interpretable. Park et al. [23] recruited women screened for distress, whose baseline scale total of 16.1 sat above the threshold at which psycho-oncological support is usually indicated, whereas Johannsen et al. [25] recruited on the basis of pain, not distress, and delivered a programme adapted to pain rather than to mood. As a contextual benchmark only, reference is made to the minimal important difference of approximately 1.7 points estimated for the scale total score in chronic obstructive pulmonary disease [18], a value not established in breast cancer. The pooled point estimate corresponds to about three times that difference, whereas the interval runs from about six times it to no effect at all; the data are compatible both with a potentially important reduction in distress and with none.

### 3.6. Anxiety

**Figure 3:**
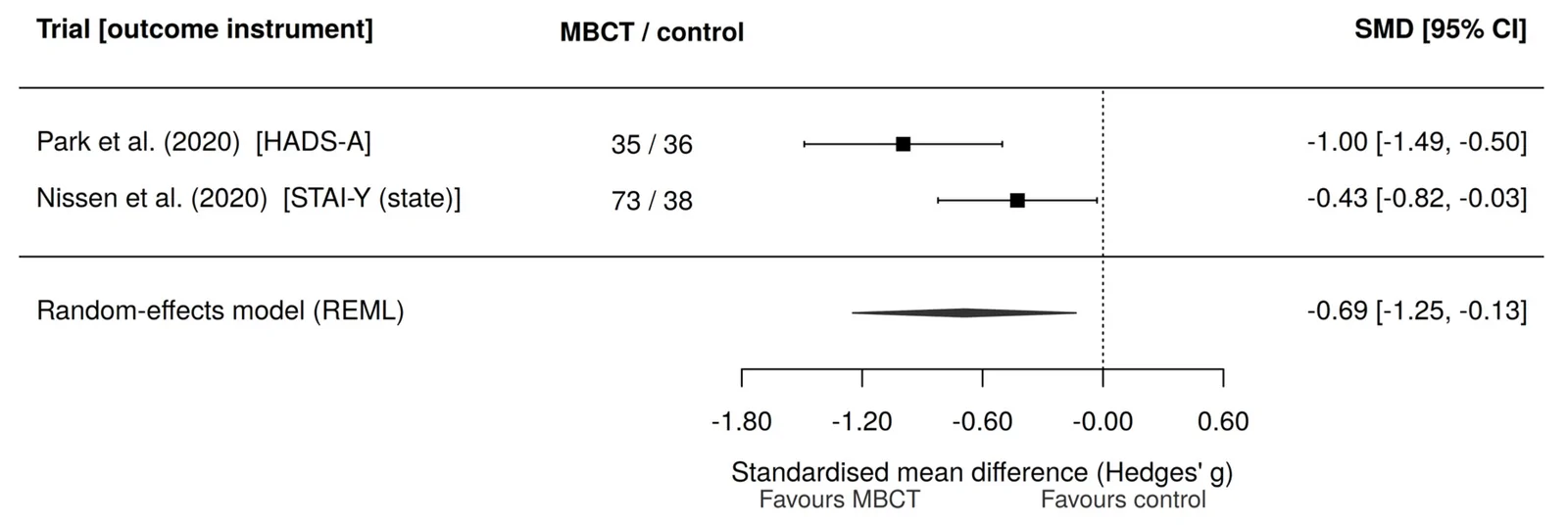
Forest plot of the association between mindfulness-based cognitive therapy and anxiety. Standardised mean differences (Hedges’ g), random-effects model, restricted maximum likelihood. Instruments differed between trials and are shown alongside each estimate [12,23].

Two trials contributed anxiety data (182 participants) using different instruments, so effects were standardised. Mindfulness-based cognitive therapy was associated with a moderate-to-large reduction in anxiety (g = −0.69, 95% CI −1.25 to −0.13; Figure 3). The trial estimates were −1.00 (95% CI −1.49 to −0.50) in Park et al. [23], measured on the anxiety subscale of the Hospital Anxiety and Depression Scale, and −0.43 (95% CI −0.82 to −0.03) in Nissen et al. [12], measured on the state subscale of the State–Trait Anxiety Inventory. Heterogeneity was moderate (*I*^2^ = 68%, *τ*^2^ = 0.110, *Q* = 3.11, *p* = 0.078); the two confidence intervals overlap and both estimates favour the intervention. Two qualifications apply. The pooled estimate does not exclude no effect when uncertainty is quantified with the Hartung–Knapp adjustment (Section 3.9), and it is therefore preliminary. It is also not derived exclusively from women with breast cancer: the estimate contributed by Nissen et al. [12] is a whole-trial estimate, and 13 of the 150 participants randomised in that trial (8.7%) were men treated for prostate cancer, a number that cannot be recovered for the subset contributing to this outcome because results are not reported by tumour type.

### 3.7. Depressive Symptoms

**Figure 4:**
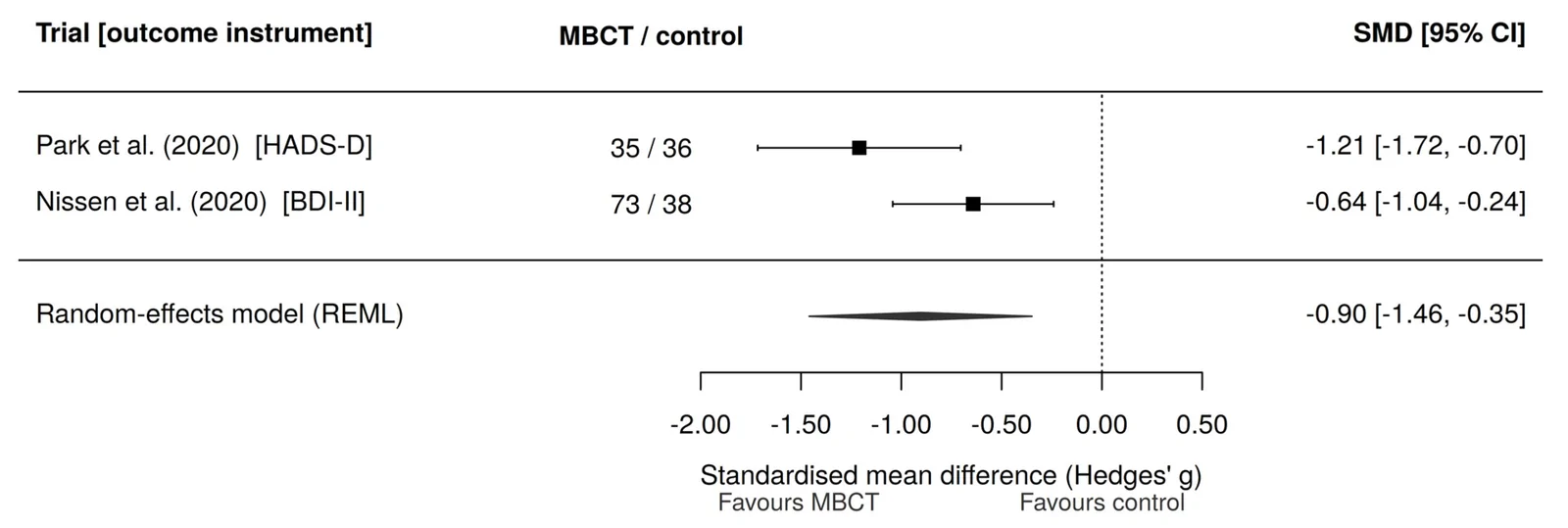
Forest plot of the association between mindfulness-based cognitive therapy and depressive symptoms. Standardised mean differences (Hedges’ g), random-effects model, restricted maximum likelihood. Instruments differed between trials and are shown alongside each estimate [12,23].

The same two trials contributed depressive symptom data (182 participants). The pooled estimate was large (g = −0.90, 95% CI −1.46 to −0.35; Figure 4), with trial estimates of −1.21 (95% CI −1.72 to −0.70) on the depression subscale of the Hospital Anxiety and Depression Scale and −0.64 (95% CI −1.04 to −0.24) on the Beck Depression Inventory-II. Heterogeneity was moderate (*I*^2^ = 66%, *τ*^2^ = 0.107, *Q* = 2.97, *p* = 0.085). As for anxiety, this estimate does not exclude no effect under the Hartung–Knapp adjustment (Section 3.9) and incorporates whole-trial data from Nissen et al. [12]; it is therefore preliminary and is not derived exclusively from women with breast cancer.

### 3.8. Fear of Cancer Recurrence

Only one trial measured fear of cancer recurrence with a validated instrument. Park et al. [23] reported a post-intervention difference of −2.16 points on the Concerns About Recurrence Scale overall subscale (95% CI −4.51 to 0.19), which the trial’s own mixed-model analysis rendered statistically significant (*p* = 0.033) with a small effect size (d = 0.43). With a single trial, no pooled estimate is possible, and the direction of effect should be regarded as suggestive only.

### 3.9. Sensitivity Analyses

Fixed-effect inverse-variance estimates were consistent in direction with the primary analyses and, as expected, narrower: −4.35 points (95% CI −6.13 to −2.58) for distress, g = −0.65 (95% CI −0.96 to −0.34) for anxiety and g = −0.86 (95% CI −1.18 to −0.55) for depressive symptoms. Hartung–Knapp intervals, computed on one degree of freedom, spanned implausibly wide ranges and included no effect for every outcome (Table 4). We report them not because they are informative in themselves but because they quantify honestly how little the two-trial evidence base constrains the estimate. The distance between the fixed-effect and Hartung–Knapp intervals for the same data is, in our view, the single most important number in this review. Restricting the synthesis to trials that enrolled women with breast cancer exclusively removed Nissen et al. [12] and reduced both the anxiety and the depressive-symptom outcomes to the single estimate of Park et al. [23]: g = −1.00 (95% CI −1.49 to −0.50) for anxiety and g = −1.21 (95% CI −1.72 to −0.70) for depressive symptoms. The direction and the approximate magnitude of both associations are preserved, and are if anything larger, but neither remains a meta-analytic estimate. The distress outcome is unaffected, because Nissen et al. [12] did not report the scale total score. The practical consequence is that the two outcomes on which this review principally rests are informed by two trials only if a trial whose randomised sample was 91.3% women with breast cancer is admitted, and by one trial otherwise; this is reflected in the certainty ratings in Section 3.10.

### 3.10. Certainty of Evidence

Certainty was rated very low for all four outcomes (Table 5). Anxiety and depressive symptoms were downgraded once for risk of bias and twice for imprecision: fewer than 200 participants contributed to each of these outcomes, far below the number that would be needed to estimate effects of the size reported with useful precision, and the interval includes no effect once uncertainty is quantified with the Hartung–Knapp adjustment. Psychological distress was downgraded for risk of bias, for inconsistency and for imprecision, and fear of cancer recurrence twice for imprecision as a single-trial outcome. No outcome was downgraded for indirectness: 340 of the 353 randomised participants (96.3%) were women with breast cancer, and the one trial contributing data from participants outside that population, Nissen et al. [12], met the 90% threshold defined before data extraction, although its results are not reported separately by tumour type. We record, however, that this judgement is what allows the anxiety and depressive-symptom outcomes to be informed by two trials rather than one, and that the analysis restricted to breast cancer-only trials in Section 3.9 removes the meta-analytic basis of both.

## 4. Discussion

Across three randomised controlled trials, mindfulness-based cognitive therapy was associated with moderate-to-large short-term reductions in anxiety and depressive symptoms in women with breast cancer. Its effect on total psychological distress and on fear of cancer recurrence could not be established with the available data. The certainty of the evidence was very low for all four outcomes, and the dominant reason was not the quality of the individual trials but their number: after excluding a suspected redundant report, no outcome in this field is informed by more than two randomised comparisons. The two associations that reached conventional significance are preliminary in a specific and testable sense: neither survived the substitution of the Hartung–Knapp interval for the conventional Wald interval, and neither remained a meta-analytic estimate when the one trial with a mixed cancer population was removed.

### 4.1. Relation to Existing Evidence

Our estimates align closely with the wider literature once that literature is stratified by programme. Ajele and Idemudia [10], synthesising 45 trials in adults with cancer, reported a pooled effect of g = −0.68 for mindfulness-based cognitive therapy specifically, against g = −1.57 for modified mindfulness programmes; our anxiety estimate of −0.69 is almost identical to theirs, and our depression estimate of −0.90 sits between their programme-specific and overall figures. Chang et al. [26], in the only previous meta-analysis restricted to mindfulness-based cognitive therapy in breast cancer, reported standardised effects of −0.70 for anxiety and −0.65 for depression. The convergence of three analyses on an effect of roughly two-thirds of a standard deviation is consistent with a real association of about that size, but it is weaker evidence than it appears: the same small trials contribute to each of these syntheses, so agreement between them is not independent replication. A further review, published while this manuscript was under consideration, synthesised 13 studies in 766 women with cancer and reported improvements in quality of life and in selected mental health outcomes, most consistently for fatigue, fear of cancer recurrence and mindfulness, with the certainty of evidence rated low to very low by its authors [27]. Its larger study count reflects a broader question: it admitted non-randomised designs, women with cancers other than breast cancer, and outcomes beyond the four considered here. The two reviews therefore bracket the same problem rather than compete, and they converge on the same verdict about the strength of the evidence, if not on the same estimates.

Where our findings diverge from earlier syntheses is on total psychological distress, and the divergence is instructive. Pooling a distress-selected sample [23] with a pain-selected sample [25] produces an estimate that describes neither. This is a limitation of the available trials, not an argument against the intervention: the effect of a mood-focused programme should be expected to depend on whether the women receiving it were selected for mood symptoms in the first place. Reviews of mindfulness in breast cancer that report improvements in quality of life of a similar order [28] face the same structural problem.

### 4.2. Implications for Personalised Supportive Care

For clinical practice, the question is not whether mindfulness-based cognitive therapy is beneficial in principle but whether it is a defensible use of scarce resources for a particular patient within a stepped and stratified model of supportive care. Three considerations follow. Each is framed as a hypothesis for prospective evaluation rather than as a recommendation supported by this meta-analysis: none derives from a formal test of moderation, and all rest on comparisons between individual trials that differ in more respects than the one under discussion.

First, the outcomes on which the evidence is strongest—anxiety and depressive symptoms—are precisely the outcomes captured by routine distress screening at follow-up visits. A programme with a plausible effect on those symptoms would, if that effect is confirmed, sit plausibly at the second step of a stepped-care pathway, after routine screening and psychoeducation and before referral for individual psychotherapy. This placement is a service-design hypothesis: no trial in this review compared the programme with either a lower-intensity or a higher-intensity alternative. Second, the reported facilitator mix raises the possibility that delivery does not require a psychologist for every session: facilitators in Park et al. [23] included psychiatrists and nurses, and in Nissen et al. [12] the internet-delivered programme was supported largely by supervised trainees. Facilitator discipline was neither randomised nor compared in any trial, so this is an observation about how three programmes happened to be staffed rather than evidence about who can deliver them. Digital mindfulness programmes in breast cancer are already being evaluated with cost-effectiveness endpoints [6]. Third, and against those two points, the evidence is thin enough that the programme should be presented to women as promising rather than established, and that services adopting it should collect their own outcome data rather than assume the published effect will transfer.

The most directly personalisable signal in our results is the divergence between the two distress estimates. The larger effect arose in the trial that recruited women screened for distress, and the smaller effect arose in the trial that recruited women based on pain; the intervention did more where the target symptom was present at entry. This is a familiar pattern in psychological medicine and an argument for symptom-based, rather than diagnosis-based, allocation to mindfulness programmes: baseline distress screening is cheap, is already performed in most survivorship services and appears to identify the phenotype in which the programme performs best. It remains, however, a between-trial comparison of two studies that differed in recruitment route, outcome instrument and intervention adaptation as well as in baseline distress, and it is therefore confounded by design. It cannot identify a patient phenotype, and we present it as the hypothesis most worth testing with individual participant data rather than as a finding.

Internet delivery deserves particular attention in services where geography or workload limits access to group programmes. The single internet-delivered trial in this review produced a smaller effect than the face-to-face trial, but it also recruited through systematic screening rather than self-referral, which plausibly enrolled less motivated participants; almost half of those allocated to the programme did not complete it. Adherence, not efficacy, may be the binding constraint on digital delivery, and adherence is a modifiable target that implementation can be designed around. Because only one internet-delivered trial was available, and because it differed from the face-to-face trials in population, recruitment route and country as well as in delivery format, no comparison between formats is supported by these data; the observation is reported to motivate a head-to-head trial, not to guide a choice between formats.

### 4.3. Implications for Research

The most consequential finding of this review for future research is procedural. A suspected redundant report, whose data are largely numerically identical to those of an included trial, circulated undetected within the evidence base, and, if both reports describe the same participants, pooling it alongside the original would double-count 74 women and inflate the apparent precision of every estimate it entered. Cell-by-cell comparison of shared instruments across candidate reports takes minutes and should be a routine step in review methodology, not an incidental discovery. Editors and peer reviewers, who are explicitly asked to compare reported results against protocols and prior publications [29], are well placed to require it.

Substantively, three priorities emerge. Trials should be adequately powered and prospectively registered; they should include active psychological comparators rather than waiting lists, so that the specific contribution of mindfulness training can be separated from attention and group contact; and they should adopt a common core outcome set, since the trials examined here used five different instruments across four constructs and thereby forced standardisation where mean differences would have been more useful. Follow-up beyond six months remains almost entirely unexamined in breast cancer, although work in mixed cancer populations now extends to nine months [30]. Finally, head-to-head comparison of face-to-face and internet delivery would answer a question that services are currently deciding on without evidence. One further observation from the updated search belongs here. Twenty of the 27 records that the citation databases added beyond PubMed/MEDLINE were published in journals not indexed in MEDLINE, and every report among them that evaluated mindfulness-based cognitive therapy in women with breast cancer was either not randomised or measured constructs other than the four considered here. The programme-specific literature is therefore larger than a count of three trials suggests, but it is largely not randomised, and it is concentrated in settings that the major indexes do not cover. A field that wants a defensible estimate needs randomised trials conducted in those settings, not more single-group or quasi-experimental studies.

### 4.4. Limitations

Three trials constitute a small evidence base, and every outcome rests on at most two of them. Confidence intervals are correspondingly wide, and the Hartung–Knapp analyses show that the data are compatible with a range of effects extending well beyond what the reference estimates suggest. We regard this inferential fragility as a substantive limitation rather than a technical footnote: under the estimator we report for comparability, two outcomes reach conventional significance, and under the estimator that is generally preferred when studies are few, none does. We have not resolved the disagreement, because it cannot be resolved with two trials per outcome.

The bibliographic search, originally completed on 4 November 2024, was updated on 17 September 2026 in PubMed/MEDLINE, Scopus and Web of Science Core Collection, and identified no further eligible trial (Section 2.3). The update is incomplete in one respect: Embase, searched in the original search, could not be included in the update. A re-run on the same date retrieved 76 records that the platform would not export, and institutional access to Embase ended before they could be screened by another route. Because Embase indexes the whole of MEDLINE, records in MEDLINE-indexed journals were covered through PubMed/MEDLINE, and Scopus covers a substantial part of the remaining journal literature; the strategy also retrieved all three included trials in both Scopus and Web of Science Core Collection. Records unique to Embase, notably conference abstracts and records from journals indexed by none of the other databases, were not examined, and an eligible trial indexed there alone could have been missed. Because no outcome is informed by more than two trials, a single additional trial could change the pooled estimates and the certainty ratings, and the findings should be read with that possibility in mind. The residual risk of a missed trial is concentrated in that fraction, and beyond it in trials not indexed in these four databases, in trial registries and conference proceedings, which we did not search systematically, and in grey literature.

Standardised mean differences were necessary because the trials used different instruments for anxiety and depressive symptoms. Standardisation assumes that the underlying constructs are equivalent across instruments and that between-participant variability is comparable, assumptions that are reasonable but not verifiable here. Two trials also selected participants based on characteristics other than the outcomes we analysed—pain in one, screening-detected anxiety or depressive symptoms in another—which limits comparability. Nissen et al. [12] included 13 men with prostate cancer whose data could not be separated from those of the 137 women with breast cancer. Retaining that trial under the 90% threshold defined before data extraction is what allows the anxiety and depressive-symptom outcomes to be pooled at all; excluding it leaves both with a single trial (Section 3.9). Readers who regard the threshold as too permissive should read the restricted estimates in Section 3.9 as the primary result and both outcomes as single-trial evidence. The anxiety and depressive-symptom outcomes also drew in part on the anxiety and depression subscales of the Hospital Anxiety and Depression Scale, which do not separate the two constructs well: the covariance structure of that scale is dominated by a general distress factor, which is why the total score is recommended for general distress [17]. Two of the four outcomes reported here are therefore less distinct from one another than their labels suggest, and the anxiety and depressive-symptom estimates contributed by Park et al. [23] should not be read as independent. All outcomes were self-reported and no trial blinded participants, which is unavoidable for a group psychological intervention but inflates effects in waiting-list comparisons. Finally, the review protocol was not prospectively registered, so the possibility of post hoc changes to the review question cannot be formally excluded; the eligibility criteria, outcomes and analysis plan reported here were, however, defined before data extraction began, and we describe them throughout as defined before data extraction rather than as prespecified, since they were not documented in a publicly accessible protocol.

## 5. Conclusions

Mindfulness-based cognitive therapy is associated with moderate-to-large short-term reductions in anxiety and depressive symptoms in women with breast cancer, but each of these associations rests on two trials, is not robust to the method used to quantify uncertainty and is of very low certainty; its association with total psychological distress and with fear of cancer recurrence remains undetermined. The certainty of this evidence is very low for all four outcomes, and the limiting factor is the number of trials rather than their conduct: three randomised comparisons, none of them designed primarily around the outcomes of greatest interest to personalised supportive care. Decisions to introduce the programme within stepped supportive care should recognise the preliminary nature of this evidence and should ideally be accompanied by prospective evaluation of outcomes in the services that adopt it. The one signal that bears directly on personalisation—that the programme performed best where the target symptom was present at entry—is hypothesis-generating and needs to be tested with individual participant data. The review also shows how easily duplicated data enter a small evidence base, and how much of the apparent weight of that evidence could rest on a single trial if its data were counted twice.

## Figures and Tables

**Table 3:** Risk of bias in the included trials, assessed with the Cochrane Risk of Bias 2 tool for the outcomes contributing to this review.

Trial	D1 Randomisation Process	D2 Deviations from Intended Interventions	D3 Missing Outcome Data	D4 Measurement of the Outcome	D5 Selection of the Reported Result	Overall
Park et al. [23]	Low	Some concerns	Low	Some concerns	Some concerns	Some concerns
Johannsen et al. [25]	Some concerns	Some concerns	High	Some concerns	Low	High
Nissen et al. [12]	Some concerns	Some concerns	Some concerns	Some concerns	Low	Some concerns

D1: Park et al. [23] used computer-generated block randomisation managed by an independent clinical research centre; Johannsen et al. [25] reported random allocation but a baseline imbalance in pain burden; Nissen et al. [12] used stratified computerised randomisation with an unbalanced 7:3 ratio and a sequence generated by the primary investigator. D2: participants could not be blinded to any of the three programmes; all trials were analysed by intention to treat with mixed models. D3: Johannsen et al. [25] had 31.3% versus 1.6% non-return of post-intervention questionnaires; Nissen et al. [12] had 26–31% versus 17% attrition with zero-effect-from-baseline sensitivity analyses. D4: all outcomes were self-reported by unblinded participants against a waiting-list comparator. D5: Nissen et al. [12] was prospectively registered (NCT03100981); Park et al. [23] reported no protocol registration. Full justifications are given in File S5.

**Table 4:** Pooled effect estimates and sensitivity analyses defined before data extraction.

Outcome	Trials	Participants	Random-Effects, Wald 95% CI (Reference)	Random-Effects, Hartung–Knapp 95% CI	Fixed-Effect 95% CI	Breast Cancer-only Trials, 95% CI
Psychological distress (HADS total), MD	2	178	−5.09 (−10.20 to 0.02); *p* = 0.051	−5.09 (−38.22 to 28.04); *p* = 0.301	−4.35 (−6.13 to −2.58)	−5.09 (−10.20 to 0.02); unchanged
Anxiety, SMD (Hedges’ g)	2	182	−0.69 (−1.25 to −0.13); *p* = 0.015	−0.69 (−4.30 to 2.92); *p* = 0.248	−0.65 (−0.96 to −0.34)	−1.00 (−1.49 to −0.50); one trial
Depressive symptoms, SMD (Hedges’ g)	2	182	−0.90 (−1.46 to −0.35); *p* = 0.001	−0.90 (−4.50 to 2.69); *p* = 0.193	−0.86 (−1.18 to −0.55)	−1.21 (−1.72 to −0.70); one trial
Fear of cancer recurrence (CARS), MD	1	71	−2.16 (−4.51 to 0.19)	Not applicable	Not applicable	−2.16 (−4.51 to 0.19); unchanged

CARS, Concerns About Recurrence Scale; CI, confidence interval; HADS, Hospital Anxiety and Depression Scale; MD, mean difference; SMD, standardised mean difference. Negative values favour mindfulness-based cognitive therapy. Heterogeneity: distress *I^2^* = 86.5%, *τ^2^* = 11.78, *Q* = 7.40, *p* = 0.007; anxiety *I^2^* = 67.8%, *τ^2^* = 0.110, *Q* = 3.11, *p* = 0.078; depressive symptoms *I^2^* = 66.4%, *τ^2^* = 0.107, *Q* = 2.97, *p* = 0.085. With two trials per outcome, the Hartung–Knapp interval is computed on one degree of freedom and is correspondingly wide; it is reported to convey the true uncertainty of the estimate rather than as the preferred summary. Prediction intervals are not reported because they are not interpretable with two studies. The final column reports the analysis restricted to trials enrolling women with breast cancer exclusively; for anxiety and depressive symptoms, this removes Nissen et al. [12] and leaves the single estimate of Park et al. [23], shown therefore as a single-trial estimate and not as a pooled effect. For contextual reference only, a minimal important difference of approximately 1.7 points was estimated for the Hospital Anxiety and Depression Scale total score in patients with chronic obstructive pulmonary disease [18]; no breast cancer-specific value is available.

**Table 5:** GRADE summary of findings: mindfulness-based cognitive therapy compared with usual care or waiting list for women with breast cancer.

Outcome (Instrument)	Trials/Participants	Effect (95% CI)	Risk of Bias	Inconsistency	Imprecision	Certainty
Anxiety (HADS-A; STAI-Y state)	2/182	SMD −0.69 (−1.25 to −0.13)	Serious	Not serious	Very serious	⊕○○○ Very low
Depressive symptoms (HADS-D; BDI-II)	2/182	SMD −0.90 (−1.46 to −0.35)	Serious	Not serious	Very serious	⊕○○○ Very low
Psychological distress (HADS total)	2/178	MD −5.09 (−10.20 to 0.02)	Serious	Serious	Serious	⊕○○○ Very low
Fear of cancer recurrence (CARS)	1/71	MD −2.16 (−4.51 to 0.19)	Serious	Not assessable	Very serious	⊕○○○ Very low

BDI-II, Beck Depression Inventory-II; CARS, Concerns About Recurrence Scale; CI, confidence interval; HADS, Hospital Anxiety and Depression Scale; HADS-A, anxiety subscale; HADS-D, depression subscale; MD, mean difference; SMD, standardised mean difference; STAI-Y, State–Trait Anxiety Inventory Y-form. Risk of bias was downgraded once because all outcomes were self-reported by unblinded participants against waiting-list comparators, and one contributing trial was at high risk of bias for missing outcome data. Inconsistency was downgraded for psychological distress because the two trial estimates do not overlap. Imprecision was downgraded twice for anxiety and for depressive symptoms, because fewer than 200 participants contributed to each of these outcomes and the interval includes no effect under the Hartung–Knapp adjustment; once for psychological distress; and twice for fear of cancer recurrence as a single-trial outcome. Indirectness was considered but not downgraded because 96.3% of randomised participants were women with breast cancer, and the one trial contributing other participants met the 90% threshold defined before data extraction; publication bias was not assessed because fewer than 10 trials contributed to any outcome.

## Data Availability

All data analysed in this review were extracted from previously published articles, which are cited in the reference list. The extracted data set, the provenance of every extracted value and the full analysis script, with the complete output of every model, are provided as Supplementary Materials (Files S3 and S6). No new primary data were generated.

## Supplementary Materials

The following supporting information can be downloaded at: https://www.mdpi.com/article/10.3390/jpm16100515/s1, File S1: Completed PRISMA 2020 checklist; File S2: Full search strategies for each database, with records screened, full texts assessed and reasons for exclusion at the updated search; File S3: Extracted data set with the table and page of provenance for every value; File S4: Cell-by-cell comparison between the excluded suspected redundant report and Park et al. [23]; File S5: Domain-level Risk of Bias 2 justifications; File S6: R analysis script and full model output.

## Abbreviations

The following abbreviations are used in this manuscript:

BDI-II	Beck Depression Inventory-II
CARS	Concerns About Recurrence Scale
CI	Confidence interval
GRADE	Grading of Recommendations, Assessment, Development and Evaluations
HADS	Hospital Anxiety and Depression Scale
iMBCT	Internet-delivered mindfulness-based cognitive therapy
MBCT	Mindfulness-based cognitive therapy
MD	Mean difference
MID	Minimal important difference
PRISMA	Preferred Reporting Items for Systematic Reviews and Meta-Analyses
RCT	Randomised controlled trial
RoB 2	Cochrane Risk of Bias 2 tool
SMD	Standardised mean difference
STAI	State–Trait Anxiety Inventory
TIDieR	Template for Intervention Description and Replication
