# Supplementary material for: Mindfulness-Based Cognitive Therapy for Anxiety, Depressive Symptoms and Psychological Distress in Women with Breast Cancer: A Systematic Review and Meta-Analysis of Randomised Controlled Trials
Source: J Pers Med. 2026 Sep 30;16(10):515. doi: 10.3390/jpm16100515 (PMC13706138; doi:10.3390/jpm16100515)
Supplement: Supplementary file 1 [file jpm-16-00515-s001.zip › jpm-4543373-supplementary.pdf]

Supplementary Material

Mindfulness-based cognitive therapy for anxiety, depressive symptoms and psychological distress in women with breast cancer: a systematic review and meta-analysis of randomised controlled trials

Contents

File S1 PRISMA 2020 checklist

File S2 Database search strategies

File S3 Extracted data with provenance of every value

File S4 Documentation of the redundant report excluded at full-text screening

File S5 Risk of bias: domain-level justifications

File S6 Analysis script

Supplementary Material File S1 — PRISMA 2020 checklist

This systematic review and meta-analysis was conducted and reported in accordance with the Preferred Reporting Items for Systematic Reviews and Meta-Analyses (PRISMA) 2020 statement (Page et al., 2021). Section numbers refer to the main manuscript.

Section / topic	Item	Checklist item (abbreviated)	Location in the manuscript
Title	1	Identify the report as a systematic review.	Title
Abstract	2	See the PRISMA 2020 for Abstracts checklist.	Abstract (structured; includes Registration)
Rationale	3	Describe the rationale for the review.	Section 1, paragraphs 1–4
Objectives	4	Provide an explicit statement of the objectives.	Section 1, final paragraph; Abstract, Objective
Eligibility criteria	5	Specify inclusion and exclusion criteria.	Section 2.2
Information sources	6	Specify all databases and other sources searched.	Section 2.3; Supplementary Material S2
Search strategy	7	Present the full search strategies.	Supplementary Material S2
Selection process	8	Specify the methods used to decide eligibility.	Section 2.4
Data collection process	9	Specify the methods used to collect data.	Section 2.5; Supplementary Material S3
Data items	10a, 10b	List and define all outcomes and other variables.	Sections 2.5 and 2.6
Study risk of bias assessment	11	Specify the methods used to assess risk of bias.	Section 2.7
Effect measures	12	Specify the effect measures used.	Section 2.8
Synthesis methods	13a–13f	Describe processes for synthesis, heterogeneity, sensitivity analyses.	Section 2.8
Reporting bias assessment	14	Describe methods to assess risk of bias due to missing results.	Section 2.8 (not assessed; fewer than 10 trials)
Certainty assessment	15	Describe methods used to assess certainty.	Section 2.9
Study selection	16a, 16b	Report numbers screened, assessed and included; cite excluded studies.	Section 3.1; Fig. 1; Section 3.3
Study characteristics	17	Cite each included study and present its characteristics.	Section 3.2; Tables 1 and 2
Risk of bias in studies	18	Present assessments of risk of bias for each study.	Section 3.4; Table 3; Supplementary Material S5
Results of individual studies	19	Present per-study summary statistics and effect estimates.	Figs. 2–4; Supplementary Material S3
Results of syntheses	20a–20d	Summarise characteristics, present each synthesis, heterogeneity and sensitivity analyses.	Sections 3.5–3.9; Table 4
Reporting biases	21	Present assessments of risk of bias due to missing results.	Section 2.8 (not assessed)
Certainty of evidence	22	Present assessments of certainty for each outcome.	Section 3.10; Table 5
Discussion	23a–23d	Provide a general interpretation, limitations and implications.	Sections 4 to 4.4
Registration and protocol	24a–24c	Provide registration information or state that the review was not registered.	Section 2.1 [TO COMPLETE]
Support	25	Describe sources of financial or non-financial support.	Funding

Section / topic	Item	Checklist item (abbreviated)	Location in the manuscript
Competing interests	26	Declare any competing interests.	Declaration of competing interest
Availability of data, code	27	Report which materials are publicly available.	Data availability; Supplementary Material S3 and S6

Supplementary Material File S2 — Search strategies and study selection: addendum for the updated search

Mindfulness-Based Cognitive Therapy for Anxiety, Depressive Symptoms and Psychological Distress in Women with Breast Cancer: A Systematic Review and Meta-Analysis of Randomised Controlled Trials.

This addendum documents the updated search of 17 September 2026. It is intended to follow the strategies of the original search (to 4 November 2024) already reported in Table S2; the original section is unchanged.

S2.A Strategies used at the update

The question has two variables, population and intervention. The comparator was deliberately omitted to maximise sensitivity, so the strategy has two blocks. No truncation or wildcards were used: every term was entered in full. Date limits were applied from each interface rather than written into the strategy.

Block P (population)

"breast cancer" OR "breast neoplasms" OR "breast carcinoma" OR "breast tumour" OR "breast tumor" OR "breast cancer survivors"

Block I (intervention)

"mindfulness-based cognitive therapy" OR "mindfulness based cognitive therapy" OR "MBCT" OR "internet-delivered mindfulness-based cognitive therapy" OR "iMBCT"

PubMed/MEDLINE — run 17 September 2026

(Block P) AND (Block I)
No field tags or brackets in the query string; limits applied from the interface: Date - Entry, 1 October 2024 to 17 September 2026.

Scopus — Advanced search, run 17 September 2026

TITLE-ABS-KEY(Block P) AND TITLE-ABS-KEY(Block I)
Run without a date limit, then restricted to records published in 2025 or 2026, i.e. after the original search closed.

Web of Science Core Collection — Advanced search, run 17 September 2026

TS=(Block P) AND TS=(Block I)
Run without a date limit, then restricted to records published in 2025 or 2026, i.e. after the original search closed.

Embase — Advanced search, run 17 September 2026

(Block P):ti,ab,kw AND (Block I):ti,ab,kw
Emtree mapping not applied. Re-run on the same date; see S2.D.

Scopus and Web of Science were deliberately re-run without a date limit before being restricted, so that the strategy could be checked against the included trials. Both retrieve Park et al. and Nissen et al., which is a validity check on the strategy itself and not only on the update.

S2.B Yield and flow of records at the update

Step	n

Records identified, PubMed/MEDLINE (entry-date window)	51
Records identified, Scopus (all years)	95
Records identified, Web of Science Core Collection (all years)	78
Records identified, three databases combined	224
Scopus and Web of Science records published 2025–2026, raw	47
duplicated between Scopus and Web of Science	10
unique across Scopus and Web of Science	37
already among the 51 PubMed/MEDLINE records (checked by DOI to PMID)	10
added by Scopus and Web of Science beyond PubMed/MEDLINE	27
Records removed before screening (duplicates, and records published before the original search closed)	146
Records screened by title and abstract	78
Records excluded at title and abstract	72
Full-text reports assessed for eligibility	6
Reports excluded at full text	6
New trials included	0

Twenty of the 27 records added by the two citation databases were published in journals not indexed in MEDLINE.
The Scopus and Web of Science export files are archived and available on request, so every count above is reproducible from the raw records.

S2.C Full-text reports assessed at the update, with reasons for exclusion

Report	Source	Reason for exclusion
Randomised trial of group MBCT versus usual care in 45 women after mastectomy. J Cancer Surviv 2026. doi:10.1007/s11764-026-02069-3	PubMed/MEDLINE	No eligible review outcome: sexual self-esteem only
Mediators and moderators of MBCT versus supportive-expressive sex education therapy in 116 breast cancer survivors. J Sex Res 2025. doi:10.1080/00224499.2025.2550057	PubMed/MEDLINE, Scopus, Web of Science	Ineligible comparator: an active psychological intervention
MBCT and parenting anxiety in 249 patients with breast cancer. World J Psychiatry 2025. doi:10.5498/wjp.v15.i12.111320	PubMed/MEDLINE, Web of Science	Non-randomised: allocation by the treatment the patient chose
MBCT on disease perception, body image and fear of cancer recurrence in women with breast cancer; three groups of 15. Sadra Med Sci J 2025. doi:10.30476/smsj.2025.101094.1471	Scopus	Non-randomised: described as quasi-experimental, no randomisation procedure reported. Reports fear of cancer recurrence

MBCT on fear of cancer progression and post-traumatic growth in 30 women with breast cancer. Int J Body Mind Culture 2025. doi:10.61838/ijbmc.v12i2.940	Scopus	Non-randomised (quasi-experimental design) and outcome is fear of progression, not fear of cancer recurrence
Online MBCT on pain severity and quality of life in 54 women receiving treatment for breast cancer. Iran J Breast Dis 2025. doi:10.61186/ijbd.17.4.96	Scopus	No eligible review outcome: pain intensity and quality of life only. Design, population, intervention and comparator were eligible

Two further reports of MBCT in women with breast cancer were excluded at title and abstract for having no eligible review outcome (death-related distress, psychological hardiness and unspecified psychological symptoms; and coping, self-compassion and empowerment), and one for an ineligible population (cyclical mastalgia rather than breast cancer).

One report retrieved by the update should have been retrieved by the original search: a 2024 quasi-experimental study of MBCT against psychoeducation in 30 patients reporting the anxiety subscale of the Hospital Anxiety and Depression Scale (Asian Pac J Cancer Prev 2024;25:3081). It is ineligible on two counts, non-randomised allocation and an active comparator, and therefore does not affect the synthesis.

S2.D Embase at the update

Embase was re-run on 17 September 2026 with the strategy above and retrieved 76 records. The platform available to the review team would not export them, and they could therefore not be de-duplicated against the other three sets or screened. Those 76 records are consequently not counted in S2.B or in Figure 1, because counting records that were never screened would misstate what was done.

Because Embase indexes the whole of MEDLINE, most of those records will already have been screened through the PubMed/MEDLINE arm of the update. The fraction unique to Embase was not examined. This is stated in the Abstract, in Section 2.3, in Section 4.4 and in a footnote to Figure 1.

Supplementary Material File S3 — Extracted data with provenance

Every value entered into the meta-analyses is listed below together with the exact table and page of the source article from which it was taken, so that each cell can be retraced independently. Values were extracted in duplicate. Where an instrument was reported only graphically, the trial was not entered into the corresponding analysis; no value was estimated from a figure.

Trial	Outcome (instrument)	Arm	n	Mean	SD	Source in the article
Park et al. (2020)	Psychological distress (HADS total)	MBCT	35	6.18	4.83	Table 3, row 'HADS-overall, T1', p. 387; n from CONSORT flow diagram, Fig. 1, p. 385
Park et al. (2020)	Psychological distress (HADS total)	Waiting list	36	14.00	8.00	Table 3, row 'HADS-overall, T1', p. 387; n from Fig. 1, p. 385
Park et al. (2020)	Anxiety (HADS-A)	MBCT	35	3.49	2.62	Table 3, row 'HADS-anxiety, T1', p. 387
Park et al. (2020)	Anxiety (HADS-A)	Waiting list	36	6.89	3.98	Table 3, row 'HADS-anxiety, T1', p. 387
Park et al. (2020)	Depressive symptoms (HADS-D)	MBCT	35	2.48	2.82	Table 3, row 'HADS-depression, T1', p. 387
Park et al. (2020)	Depressive symptoms (HADS-D)	Waiting list	36	7.11	4.53	Table 3, row 'HADS-depression, T1', p. 387
Park et al. (2020)	Fear of cancer recurrence (CARS)	MBCT	35	10.53	4.93	Table 3, row 'FCR, T1', p. 387
Park et al. (2020)	Fear of cancer recurrence (CARS)	Waiting list	36	12.69	5.16	Table 3, row 'FCR, T1', p. 387
Johannsen et al. (2016)	Psychological distress (HADS total)	MBCT	46	15.70	5.10	Table 2, row 'HADS', column 'Post intervention, Treatment', p. 3395; n reported in the same cell
Johannsen et al. (2016)	Psychological distress (HADS total)	Waiting list	61	18.30	6.40	Table 2, row 'HADS', column 'Post intervention, Control', p. 3395; n reported in the same cell
Nissen et al. (2020)	Anxiety (STAI-Y state)	iMBCT	73	37.50	8.90	Table 1, row 'STAI-Y', column 'T3: Post-Intervention, iMBCT', p. 72
Nissen et al. (2020)	Anxiety (STAI-Y state)	Care as usual	38	41.80	11.90	Table 1, row 'STAI-Y', column 'T3: Post-Intervention, Control', p. 72
Nissen et al. (2020)	Depressive symptoms (BDI-II)	iMBCT	73	10.70	6.60	Table 1, row 'BDI-II', column 'T3: Post-Intervention, iMBCT', p. 72
Nissen et al. (2020)	Depressive symptoms (BDI-II)	Care as usual	38	15.60	9.20	Table 1, row 'BDI-II', column 'T3: Post-Intervention, Control', p. 72

Note. BDI-II, Beck Depression Inventory-II; CARS, Concerns About Recurrence Scale; HADS, Hospital Anxiety and Depression Scale; iMBCT, internet-delivered mindfulness-based cognitive therapy; SD, standard deviation; STAI-Y, State–Trait Anxiety Inventory Y-form. Johannsen et al. (2016) did not administer the Concerns About Recurrence Scale and reported the Hospital Anxiety and Depression Scale only as a total score; the trial therefore contributes to the distress analysis alone. Nissen et al. (2020) did not administer the Hospital Anxiety and Depression Scale.

Supplementary Material File S4 — Documentation of the redundant report

One report retrieved at full-text screening — Chu, X., Lou, Y., Wang, Z., Xu, Q., 2020. Effect of mindfulness based cognitive therapy on the mental health and quality of life in patients with breast cancer. Indian Journal of Pharmaceutical Sciences 82 (Special issue 7), 48–52 — was excluded as a redundant publication of Park et al. (2020).

The report describes 84 women recruited in Nanjing, China, between August 2017 and August 2018, whereas Park et al. (2020) describes 74 women recruited in Tokyo, Japan, between January 2015 and August 2016. Despite these differences, the two reports present outcome data that coincide to two decimal places across multiple instruments and time points. The comparison below lists every cell the two reports share. It is not possible for two independently conducted trials in different countries, with different sample sizes, to generate identical means and standard deviations across whole instruments.

In addition, the Results text of the report states that 'the average difference of the total score of HADS was −7.82', a value that contradicts its own table (−7.77) but reproduces exactly the between-group difference reported by Park et al. (2020). The discussion section of the report also paraphrases that of Park et al. (2020) sentence by sentence, and its reference list substantially overlaps.

The report is not indexed in PubMed; coverage of the journal in that database ends in 2016. No retraction notice could be located, and none can be excluded from PubMed alone. We make no claim about how the duplication arose. We record it because the report is retrievable through other databases and has been treated as an independent trial in at least one previous synthesis, and because pooling it with Park et al. (2020) counts the same 74 women twice.

Table S4.1. Cell-by-cell comparison of the outcome data reported by Park et al. (2020) and by Chu et al. (2020).

Instrument and time point (arm)	Park et al. (2020)	Chu et al. (2020)	Comparison
HADS total, baseline (control)	14.81 (6.99)	14.81 ± 6.99	Identical
HADS total, week 8 (control)	14.00 (8.00)	14.00 ± 8.00	Identical
HADS total, week 12 (control)	13.33 (6.97)	13.33 ± 6.97	Identical
HADS-anxiety, week 8 (intervention)	3.49 (2.62)	3.51 ± 2.62	Identical SD

Instrument and time point (arm)	Park et al. (2020)	Chu et al. (2020)	Comparison
HADS-anxiety, week 8 (control)	6.89 (3.98)	6.97 ± 3.98	Identical SD
HADS-depression, baseline (intervention)	7.84 (4.38)	7.74 ± 4.38	Identical SD
HADS-depression, week 8 (intervention)	2.48 (2.82)	2.38 ± 2.82	Identical SD
HADS-depression, week 8 (control)	7.11 (4.53)	7.33 ± 4.53	Identical SD
BFI, baseline (both arms)	3.54 (2.28) / 3.22 (2.42)	3.54 ± 2.28 / 3.22 ± 2.42	Identical
BFI, week 8 (control)	3.37 (2.81)	3.37 ± 2.81	Identical
BFI, week 12 (control)	3.04 (2.37)	3.04 ± 2.37	Identical
FACT-G, baseline (both arms)	65.20 (16.77) / 68.91 (16.63)	65.20 ± 16.77 / 68.91 ± 16.63	Identical
FACT-G, week 8 (both arms)	84.94 (14.59) / 71.59 (18.73)	84.94 ± 14.59 / 71.59 ± 18.73	Identical
FACT-G, week 12 (both arms)	85.41 (16.08) / 71.90 (18.50)	85.41 ± 16.08 / 71.90 ± 18.50	Identical
FFMQ, week 8 (both arms)	133.67 (17.87) / 120.47 (14.37)	133.67 ± 17.87 / 120.47 ± 14.37	Identical
FFMQ, week 12 (both arms)	137.03 (18.85) / 119.19 (13.44)	137.03 ± 18.85 / 119.19 ± 13.44	Identical
FACIT-Sp, week 8 (both arms)	32.70 (9.63) / 24.61 (8.25)	32.70 ± 9.64 / 24.61 ± 8.54	Near-identical
FCR / CARS, week 8 (control)	12.69 (5.16)	12.69 ± 4.78	Identical mean
Reported between-group difference, HADS total, week 8	−7.82	Table: −7.77; text: −7.82	Text value matches Park et al. (2020), not its own table

Note. BFI, Brief Fatigue Inventory; CARS, Concerns About Recurrence Scale; FACIT-Sp, Functional Assessment of Chronic Illness Therapy — Spiritual Well-Being; FACT-G, Functional Assessment of Cancer Therapy — General; FFMQ, Five Facet Mindfulness Questionnaire; HADS, Hospital Anxiety and Depression Scale. Values are mean (standard deviation). Park et al. (2020) values are taken from Tables 3; Chu et al. (2020) values from its Tables 2 to 5.

Supplementary Material File S5 — Risk of bias: domain-level justifications

Domain-level justifications for the Cochrane Risk of Bias 2 judgements summarised in Table 3 of the main manuscript. Assessments were made for the outcomes contributing to this review.

Park et al. (2020) — Overall: some concerns.

D1 (randomisation process): block randomisation with a computer-generated list, stratified by baseline anxiety, managed by an organisation independent of the research team; baseline characteristics comparable — low risk. D2 (deviations from intended interventions): blinding of participants is not feasible for a group psychological programme and no restriction was placed on co-interventions; analyses followed the intention-to-treat principle with a mixed model — some concerns. D3 (missing outcome data): three of 38 participants in the intervention arm and none in the control arm discontinued; outcome data were available for a high proportion of participants and the analysis model accommodated missingness — low risk. D4 (measurement of the outcome): all outcomes self-reported by participants aware of allocation, against a waiting-list comparator — some concerns. D5 (selection of the reported result): the primary outcome is clearly stated and all prespecified outcomes are reported, but no protocol or trial registration is cited — some concerns.

Johannsen et al. (2016) — Overall: high risk of bias.

D1 (randomisation process): random allocation to a waiting-list control is reported, but the sequence generation and concealment procedures are described only briefly and a baseline imbalance in perceived pain burden was observed and adjusted for post hoc — some concerns. D2: participants were aware of allocation; analyses were by intention to treat with mixed linear models — some concerns. D3 (missing outcome data): 31.3% of the intervention arm did not return post-intervention questionnaires compared with 1.6% of the control arm; the authors report this differential attrition explicitly and conducted sensitivity analyses for the primary pain outcome, but not for the psychological distress outcome used here — high risk. D4: self-reported outcome, unblinded participants — some concerns. D5: psychological distress is prespecified as a secondary outcome and is reported with full descriptive statistics at all time points — low risk.

Nissen et al. (2020) — Overall: some concerns.

D1 (randomisation process): computerised randomisation stratified by cancer type, but with an unbalanced 7:3 allocation and an allocation sequence generated by the primary investigator; research assistants handling recruitment were unaware of allocation until after inclusion — some concerns. D2: 49% of participants allocated to the intervention did not start or discontinued within four weeks; analyses were by intention to treat with multilevel modelling — some concerns. D3 (missing outcome data): attrition of 26–31% in the intervention arm versus about 17% in the control arm, with prespecified zero-effect-from-baseline sensitivity analyses that preserved the anxiety result and attenuated the depression result — some concerns. D4: self-reported outcomes, unblinded participants — some concerns. D5: the trial was prospectively registered (ClinicalTrials.gov NCT03100981) and both primary outcomes are reported — low risk.

Supplementary Material File S6 — Analysis script

The analysis was performed in R version 4.3.3 using the metafor package. The script below reproduces every estimate reported in the manuscript from the extracted data set given in Supplementary Material S3. The complete console output, including session information, accompanies this file as a plain-text log.

```
################################################################################
# MBCT for psychological outcomes in women with breast cancer
# Systematic review and meta-analysis - ANALYSIS v5 (rebuilt 2026-08-18)
#
# All data extracted from the post-intervention tables of the source articles.
# Provenance of every cell is documented in Supplementary Material S3.
#   Park et al. 2020,      J Pain Symptom Manage 60(2):381-389, Table 3 (T1)
#   Johannsen et al. 2016, J Clin Oncol 34(28):3390-3399,       Table 2 (post)
#   Nissen et al. 2020,    Psycho-Oncology 29(1):68-75,          Table 1 (T3)
#
# Chu et al. 2020 (Indian J Pharm Sci) EXCLUDED: redundant publication of the
# Park et al. 2020 dataset (see Supplementary Material S4).
################################################################################

suppressMessages(library(metafor))
set.seed(20260818)
OUT <- "/tmp/mb/resubmission"

## ---------------------------------------------------------------- 1. DATA ---
# Psychological distress - HADS total score (same instrument -> MD)
distress <- data.frame(
  study = c("Park et al. (2020)", "Johannsen et al. (2016)"),
  n1 = c(35, 46), m1 = c(6.18, 15.70), sd1 = c(4.83, 5.10),
  n2 = c(36, 61), m2 = c(14.00, 18.30), sd2 = c(8.00, 6.40),
  instrument = c("HADS total", "HADS total"))

# Anxiety - different instruments (HADS-A vs STAI-Y state) -> SMD
anxiety <- data.frame(
  study = c("Park et al. (2020)", "Nissen et al. (2020)"),
  n1 = c(35, 73), m1 = c(3.49, 37.50), sd1 = c(2.62, 8.90),
  n2 = c(36, 38), m2 = c(6.89, 41.80), sd2 = c(3.98, 11.90),
  instrument = c("HADS-A", "STAI-Y (state)"))

# Depressive symptoms - different instruments (HADS-D vs BDI-II) -> SMD
depression <- data.frame(
  study = c("Park et al. (2020)", "Nissen et al. (2020)"),
  n1 = c(35, 73), m1 = c(2.48, 10.70), sd1 = c(2.82, 6.60),
  n2 = c(36, 38), m2 = c(7.11, 15.60), sd2 = c(4.53, 9.20),
  instrument = c("HADS-D", "BDI-II"))

# Fear of cancer recurrence - single trial, no pooling
fcr <- data.frame(
  study = "Park et al. (2020)",
  n1 = 35, m1 = 10.53, sd1 = 4.93,
  n2 = 36, m2 = 12.69, sd2 = 5.16, instrument = "CARS (overall FCR)")

## -------------------------------------------------------- 2. EFFECT SIZES ---
es <- function(d, measure) escalc(measure = measure,
```

```r
        m1i = d$m1, sd1i = d$sd1, n1i = d$n1,
        m2i = d$m2, sd2i = d$sd2, n2i = d$n2, data = d, slab = d$study)

distress   <- es(distress,   "MD")
anxiety    <- es(anxiety,    "SMD")   # Hedges' g
depression <- es(depression, "SMD")
fcr        <- es(fcr,        "MD")

## ------------------------------------------------- 3. POOLED ESTIMATES -----
fit <- function(d) list(
  re    = rma(yi, vi, data = d, method = "REML"),                 # primary
  hk    = rma(yi, vi, data = d, method = "REML", test = "knha"),  # sensitivity
  fe    = rma(yi, vi, data = d, method = "FE"))                   # sensitivity

res <- list(distress = fit(distress), anxiety = fit(anxiety),
            depression = fit(depression))

line <- function(m, lab, dig = 2) sprintf(
  "%-42s %6.2f (%6.2f to %6.2f)  p=%.4f  I2=%4.1f%%  tau2=%.4f",
  lab, m$b, m$ci.lb, m$ci.ub, m$pval, m$I2, m$tau2)

sink(file.path(OUT, "Analysis", "analysis_output_v5.txt"))
cat("MBCT META-ANALYSIS v5 - OUTPUT\n")
cat("Generated:", format(Sys.time(), "%Y-%m-%d %H:%M:%S"), "\n")
cat("metafor", as.character(packageVersion("metafor")), "| R", R.version.string, "\n")
cat(strrep("=", 100), "\n\n")

for (nm in names(res)) {
  cat("### ", toupper(nm), "\n", sep = "")
  d <- get(nm)
  for (i in seq_len(nrow(d)))
    cat(sprintf("   %-26s %-16s  n=%3d/%3d  y=%7.3f  SE=%.3f  95%%CI %6.2f to %6.2f\n",
        d$study[i], d$instrument[i], d$n1[i], d$n2[i], d$yi[i], sqrt(d$vi[i]),
        d$yi[i]-1.96*sqrt(d$vi[i]), d$yi[i]+1.96*sqrt(d$vi[i])))
  cat("\n", line(res[[nm]]$re, "  Random-effects REML (Wald) [PRIMARY]"), "\n")
  cat(line(res[[nm]]$hk, "  Random-effects REML + Hartung-Knapp"), "\n")
  cat(line(res[[nm]]$fe, "  Fixed-effect inverse-variance"), "\n")
  cat("  Q =", round(res[[nm]]$re$QE,3), " p_Q =", round(res[[nm]]$re$QEp,4), "\n")
  cat("  Leave-one-out (k=2 -> each single trial): see individual estimates above\n\n")
}
cat("### FEAR OF CANCER RECURRENCE - single trial, not pooled\n")
cat(sprintf("   %-26s %-18s n=%d/%d  MD=%.2f  95%%CI %.2f to %.2f\n\n",
    fcr$study, fcr$instrument, fcr$n1, fcr$n2, fcr$yi,
    fcr$yi-1.96*sqrt(fcr$vi), fcr$yi+1.96*sqrt(fcr$vi)))
cat(strrep("=", 100), "\nSESSION INFO\n"); print(sessionInfo())
sink()

## -------------------------------------------------------- 4. FOREST PLOTS ---
mkforest <- function(d, m, file, xlab, header2, xlim, alim, at, digits = 2) {
  slab2 <- paste0(d$study, "  [", d$instrument, "]")
  tiff(file.path(OUT, "Figures", file), width = 9, height = 3.8,
       units = "in", res = 600, compression = "lzw")
  par(mar = c(6.6, 1, 1.8, 1), family = "sans")
  forest(m, slab = slab2, xlim = xlim, alim = alim, at = at,
         ilab = sprintf("%d / %d", d$n1, d$n2),
         ilab.xpos = xlim[1] + 0.415*(xlim[2]-xlim[1]),
         xlab = xlab, header = c("Trial [outcome instrument]", header2),
         mlab = "Random-effects model (REML)", digits = digits,
         col = "grey20", border = "grey20", cex = 0.95, psize = 1.2,
         refline = 0)
  text(xlim[1] + 0.415*(xlim[2]-xlim[1]), m$k + 2,
```

```
       "MBCT / control", font = 2, cex = 0.92)
  par(xpd = NA)
  text(alim[1], -4.35, "Favours MBCT",    pos = 4, cex = 0.86, col = "grey20")
  text(alim[2], -4.35, "Favours control", pos = 2, cex = 0.86, col = "grey20")
  par(xpd = FALSE)
  dev.off()
}

mkforest(distress, res$distress$re, "Figure2_distress.tiff",
         "Mean difference in HADS total score", "MD [95% CI]",
         xlim = c(-34, 12), alim = c(-13, 3), at = seq(-12, 3, 3))
mkforest(anxiety, res$anxiety$re, "Figure3_anxiety.tiff",
         "Standardised mean difference (Hedges' g)", "SMD [95% CI]",
         xlim = c(-5.4, 2.2), alim = c(-1.8, 0.6), at = seq(-1.8, 0.6, 0.6))
mkforest(depression, res$depression$re, "Figure4_depression.tiff",
         "Standardised mean difference (Hedges' g)", "SMD [95% CI]",
         xlim = c(-5.4, 2.2), alim = c(-2.0, 0.5), at = seq(-2.0, 0.5, 0.5))

## ------------------------------------------------------ 5. MACHINE-READABLE ---
tab <- do.call(rbind, lapply(names(res), function(nm) {
  m <- res[[nm]]
  data.frame(outcome = nm, k = m$re$k,
    n_total = sum(get(nm)$n1) + sum(get(nm)$n2),
    estimate = as.numeric(m$re$b), ci_lb = m$re$ci.lb, ci_ub = m$re$ci.ub,
    p = m$re$pval, I2 = m$re$I2, tau2 = m$re$tau2,
    hk_lb = m$hk$ci.lb, hk_ub = m$hk$ci.ub, hk_p = m$hk$pval,
    fe_est = as.numeric(m$fe$b), fe_lb = m$fe$ci.lb, fe_ub = m$fe$ci.ub)
}))
write.csv(tab, file.path(OUT, "Analysis", "pooled_estimates_v5.csv"), row.names = FALSE)
write.csv(rbind(
  cbind(outcome="distress",   distress[,c("study","instrument","n1","m1","sd1","n2","m2","sd2","yi","vi")]),
  cbind(outcome="anxiety",    anxiety[,c("study","instrument","n1","m1","sd1","n2","m2","sd2","yi","vi")]),
  cbind(outcome="depression", depression[,c("study","instrument","n1","m1","sd1","n2","m2","sd2","yi","vi")]),
  cbind(outcome="fcr",        fcr[,c("study","instrument","n1","m1","sd1","n2","m2","sd2","yi","vi")])),
  file.path(OUT, "Analysis", "extracted_data_v5.csv"), row.names = FALSE)
cat("DONE\n")
```
